# EZmito: a simple and fast tool for multiple mitogenome analyses

**DOI:** 10.1080/23802359.2021.1899865

**Published:** 2021-03-19

**Authors:** Claudio Cucini, Chiara Leo, Nicola Iannotti, Sara Boschi, Claudia Brunetti, Joan Pons, Pietro Paolo Fanciulli, Francesco Frati, Antonio Carapelli, Francesco Nardi

**Affiliations:** a Department of Life Sciences, University of Siena, Siena, Italy; b Department of Life Sciences, Imperial College London, London, UK; c Department de Biodiversitat Animal i Microbiana, Institut Mediterrani d’Estudis Avancats, Esporles, Spain

**Keywords:** Web Server, phylogeny, mitogenome, nucleotide bias, RSCU

## Abstract

Complete mitochondrial genome data are frequently applied to address phylogenetic/phylogeographic issues at different taxonomic levels in ecology and evolution. While sample preparation/sequencing is becoming more and more straightforward thanks to dropping costs for next-generation sequencing (NGS), data preparation and visualization remains a manually intensive step that may lead to errors if improperly conducted. We have elaborated, and here introduce, EZmito, a simple and intuitive, freely accessible Web Server aimed at automating some of these tasks. EZmito is divided into three main tools: EZpipe that assembles DNA matrices for phylo-mitogenomic analyses; EZskew that calculates genome, strand, and codon nucleotide compositional skews and EZcodon which computes Relative Synonymous Codon Usage statistics as well as amino acid usage frequency over multiple mitogenomes. Output is produced in tabular format as well as publication-quality graphics.

## 1. Introduction

Over time, mitochondrial genetics and genomics passed through a noticeable revolution in the process of data acquisition. Until the last decade, complete mitochondrial genome sequences were obtained using a classic PCR and Sanger sequencing approaches, with processing time per genome being as long as one year in the '90s (Nardi et al. 2001) and a month in the '00s (Nardi et al. 2010). With the advent of next-generation sequencing (NGS) technologies, processing time and associated costs dropped, leading to a substantial increase in the rate of production, annotation, and publication of new mitochondrial genomes (i.e. 11,758 in January 2021 in the NCBI Organelle Database). As of today, the time needed for analysis, and not for sequence acquisition, is the limiting factor.

The considerable simplicity in data acquisition has led evolutionary biologists to use mitochondrial multi-locus or complete mitogenome analyses to study phylogenetic relationships at different taxonomic levels thanks to: (i) improved resolution compared to single-locus analyses (i.e. Carapelli et al. 2007); (ii) informativeness at different taxonomic levels due to the presence of genes characterized by dissimilar evolutionary rates, as well as the possibility to recode data as amino acids and according to the positioning of single genes (gene order); and (iii) availability of data from other species that can be readily included due to strict orthology of the mitochondrial genome across Metazoa. Therefore, phylomitogenomics have become an important research tool in evolutionary biology at all phylogenetic levels (e.g. Finstermeier et al. 2013; Hikosaka et al. 2013). Given the increasing importance of mitogenomes for phylogenetic purposes, specialized journals (e.g. Mitochondrial DNA Part B Resources) were launched with the primary aim of publishing new mitochondrial DNA sequences to be used in phylomitogenomic analyses.

To ease mitogenome assembly and annotation of high-throughput sequencing data, new and dedicated bioinformatic tools have been developed (e.g. Bernt et al. 2013; Dierckxsens et al. 2017). At the same time, multiple options are available for sequence alignment (e.g. ClustalW, Thompson et al. 1994; MUSCLE, Edgar 2004; MAFFT, Katoh et al. 2005, etc.) and tree building (MrBayes, Ronquist et al. 2012; RAxML, Stamatakis 2014; IQ-TREE, Nguyen et al. 2015) that, although not specifically developed for mitochondrial genomes, are obviously appropriate. However, the basic steps of dataset building and visualization still require a significant amount of hands-on time as: (i) no pipeline is available that is exactly designed for mitogenome data set preparation and that, as such, takes advantage of the relative uniformity of the analyses performed (but see NGPhylogeny (Lemoine et al. 2019) and PhyloSuite (Zhang et al. 2020) for general phylogenetic/phylogenomic pipelines); (ii) these basic steps can be accomplished with any of multiple software (e.g. Aliview, Larsson 2014; Mesquite, http://www.mesquiteproject.org for concatenation and removal of third codon positions; MS Excel or R for the plotting of base composition) that are usually on the everyday toolkit of the professional phylogeneticist, thus limiting the interest in a shared automated solution. At the same time, some aspects of the analysis that are regularly applied to mitochondrial genomes, though simple, require some attention and experience to be properly performed, such as (i) an initial sanity check on the sequences; (ii) removal of hypervariable regions without disrupting the coding frame; (iii) concatenation by name across multiple datasets; and (iv) identification of a basic set of partitions to initiate the partitioning and model optimization analysis.

Following the observation that it is not uncommon to see analyses that contain basic errors in data preparation/handling, such as truncated genes, PCGs with stop codons, gaps not following the coding frame (citations voluntarily omitted), we deem that an automated procedure covering these preparatory steps, simple as it is, may save errors to the practicing and time to the experienced phylogeneticist.

A further area of interest in mitochondrial genomics, stemming from molecular evolution but being relevant for phylogenetics alike due to model assumption violation issues, includes the study of base compositional biases. Due to DNA polymerase errors during replication, the limited repair mechanisms, and highly oxidative environment of the mitochondrion, the mtDNA is inclined to directional mutations (especially deaminations) that result in whole genome or strand-specific nucleotide biases within different taxa. Following Hassanin et al. (2005), the comparison of mitogenomes of several Metazoa lineages allowed for the identification of at least three major nucleotide biases in metazoans’ mtDNAs: (i) a whole-genome sequence bias, such as the overall A-T richness frequently observed in insects; (ii) a strand bias, affecting the two strands differently (J- and N-strand in insects, corresponding to L- and H-strand in vertebrates); and (iii) a codon bias, affecting nucleotides within triplets differently. Furthermore, a codon usage imbalance is also noticeable, as exemplified by the differential use of synonymous triplets. While basic compositional bias statistics can be calculated and visualized with the help of a spreadsheet and/or graphic software, and specialized solutions are available (Puigbò et al. 2008; González-Castellano et al. 2020), obtaining good quality graphics is a time-consuming process and one that, given the uniformity of the plots employed, may greatly benefit from standardization.

In order to provide a simple and automated workflow for the community of evolutionary biologists working with complete mitochondrial genomes, we created and herein describe the EZmito pipeline. This, in the form of a freely usable Web Server (http://ezmito.unisi.it), executes the basic data preparation and visualization steps that are generally performed in the ESZ_lab (Evolutionary & Systematic Zoology laboratory) as a preparatory step for mtDNA data analyses. The Web Server is a simple and intuitive bioinformatic tool designed to: (i) assemble complete mitochondrial genome datasets for phylogenetic purposes; (ii) calculate and visualize strand, codon, and positional nucleotide biases; and (iii) calculate and visualize amino acid and codon usage, as well as the Relative Synonymous Codon Usage (RSCU) across multiple mitogenomes.

## 2. Materials and methods

EZmito is a web application coded in python, R, and bash. It is hosted on a virtual server at the University of Siena (Italy) and it is divided into three main tools: EZpipe, EZskew, and EZcodon.

### 2.1. EZpipe

EZpipe is designed to prepare mitochondrial PCGs data sets to be used in phylogenetic analyses, as seen in recent papers from our group (e.g. Leo et al. 2019; Cucini et al. 2020). In detail, the tool requires a compressed archive (.zip, .tar, .rar, .gz, .7z) of fasta files corresponding to individual PCGs (e.g. cox1.fasta, cox2.fasta, cox3.fasta, etc*.,* in separate files) containing nonaligned sequences with unique taxon names (common to all files) as sequence headers. It also takes two input parameters: the appropriate genetic code (follows NCBI designations, e.g. 5 for the invertebrate mitochondrial code) and the number of nucleotide codon positions that should be analyzed (e.g. 3 for all codon positions, 2 for first and second codon positions only).

Initially, input files undergo a sanity check to assess if the data provided are correctly formatted and suitable for downstream analyses. Warnings are displayed if: (i) duplicated sequences are found within the data set; (ii) the length of a sequence differs by more than 2 standard deviations from the mean length of all sequences (e.g. if a gene is truncated); (iii) gaps are found within sequences – in this case, they will be automatically removed; and (iv) sequences display a truncated end codon – which will be automatically removed. In all these cases, the analysis continues. On the other hand, an error is displayed if: (i) the input archive is not prepared as required (e.g. a directory is present in the compressed file); (ii) input files are not in fasta format; (iii) duplicated sequence IDs are present within a file; (iv) non-IUPAC nucleotides are observed in a sequence; and v) stop codons are present within a sequence (i.e. not counting terminal stop codons). In all these cases the analysis is stopped.

After sanity check, sequences in each file are retro-aligned using RevTrans (Wernersson and Pedersen 2003) based on the proper genetic code. Hyper-variable regions of unreliable alignment are further discarded using Gblocks (Castresana 2000), removing full codons through options *strict* and *codon,* thus respecting the coding frame. Finally, single-gene alignments are concatenated through the concatenateAlignments R function and converted in the phylip format. A PartitionFinder2 configuration file is created where a starting partitioning scheme is designed subdividing the final alignment by gene and by codon position (e.g. if all 13 PCGs are included, 39 starting partitions are created if analyzing all codon positions, 26 if analyzing first, and second codon positions only). These two latter files, the concatenated alignment in phylip format and the PartitionFinder2 configuration file, are the main output of EZpipe. Three log files are written for troubleshooting at different levels, of which the log.txt file includes information relevant for the user.

EZpipe does not include a tree-building step. Nevertheless, the two produced files can be used in downstream applications in two different ways to produce a phylogenetic tree. The concatenated alignment and the PartitionFinder2 configuration file can be directly used as input for PartitionFinder2 (Lanfear et al. 2017) to optimize the partitioning scheme and associated evolutionary models. This will further produce command blocks to be used in commonly used phylogenetic software (e.g. MrBayes, IQtree, and RAxML). As an alternative, the concatenated alignment can be directly used as input for phylogenetic analyses not requiring partitioning (not recommended).

Noteworthy, the model optimization and tree building steps are computationally intensive tasks for datasets of medium-large size (>30 genomes) and may require hardware not readily available in every laboratory, prompting for the use of external services. The CIPRES Science Gateway (Miller et al. 2010) is a public high-performance computing infrastructure devoted to phylogenetic computation. It implements and maintains all commonly used phylogenetic software (e.g. PartitionFinder2, MrBayes, and IQtree) and provides free, though limited, service plans.

### 2.2. EZskew

EZskew calculates nucleotide biases by the protein-encoding part of the genome, strand, and codon position. It takes as input a compressed archive (.zip, .tar, .rar, .gz, .7z) including two folders (J and N), each containing a fasta file of nonaligned protein-encoding genes in the expected orientation. The appropriate genetic code is set as an input parameter. An initial sanity check is performed as described in Section 2.1. Then, each PCG stack is padded with gaps and concatenated to produce three final matrices: full genome data, J-strand only, and N-strand only. Concatenated matrices are used for the calculation of compositional skews following Hassanin et al. (2005). AT%, being strand independent, is calculated over the entire PCG concatenated matrix, while AC% and GT% are calculated for PCGs over the concatenated J- and N-strands, respectively. These values are plotted in a single histogram by genome. Codon position biases, namely the AT and CG skews, are calculated over the J- and N-strand separately for first, second, and third codon positions using formulas:

$$\mathrm{AT skew}=\frac{A-T}{A+T}\;\mathrm{CG skew}=\frac{C-G}{C+G}$$

and plotted separately for each codon position. A text-based tabular output is also produced, as well as log files. In the attempt to produce publication-quality graphics, the level of detail automatically adapts to the number of genomes being analyzed (see below).

### 2.3. EZcodon

EZcodon is designed to calculate amino acid frequencies and RSCU over different genomes. It requires the same input files as EZskew. Initial steps (sanity check and concatenation) are as described for EZskew (see Section 2.2) to obtain full genome data in coding orientation. This matrix is used to calculate the frequency of each amino acid as well as the RSCU table using the CAI python package (Lee 2018). Non-conventional amino acids (i.e. translated starting from ambiguous nucleotides: R, S, etc.) are deleted during this step. Two graphical outputs are produced: (i) amino acid frequencies in genes encoded in the J- and N-strand genes, as well as all genes; and (ii) RSCU values for each genome (i.e. all codons despite the strand orientation of genes). A text-based output and log files are also produced. The level of detail automatically adapts as a function of the number of genomes being analyzed (see below).

### 2.4. Data set preparation examples

In order to display EZmito functionalities and evaluate computing time, two recently published data sets, characterized by a different number of species, were processed: data from Cucini et al. (2020), henceforth XL, includes 90 genomes; while data from Carapelli et al. (2019), henceforth S, includes 18 genomes. Both original studies included phylomitogenomic analyses and nucleotide bias calculations that, though performed manually, are grossly superimposable with EZmito analyses.

## 3. Results

The two aforementioned data sets (XL and S) were processed using the three tools EZpipe, EZskew, and EZcodon.

### 3.1. EZpipe output

Data set preparation was accomplished using EZpipe on 3 and 2 codon positions (i.e. including and excluding the third codon positions) for both datasets. The XL data set was processed in less than two minutes (101 s) whereas the smaller data set in about 7 s (Table 1).

**Table 1.** EZmito bioinformatic tools time processing.

Tool	Data set	Species number	Genetic code	Processing time (s)
Ezpipe (3 codon positions)	XL	90	5 – Invertebrate	101
	S	18	5 – Invertebrate	6
Ezpipe (2 codon positions)	XL	90	5 – Invertebrate	101
	S	18	5 – Invertebrate	7
EZskew	XL	90	5 – Invertebrate	15
	S	18	5 – Invertebrate	8
EZcodon	XL	90	5 – Invertebrate	104
	S	18	5 – Invertebrate	23

### 3.2. EZskew output

**Figure 1.**
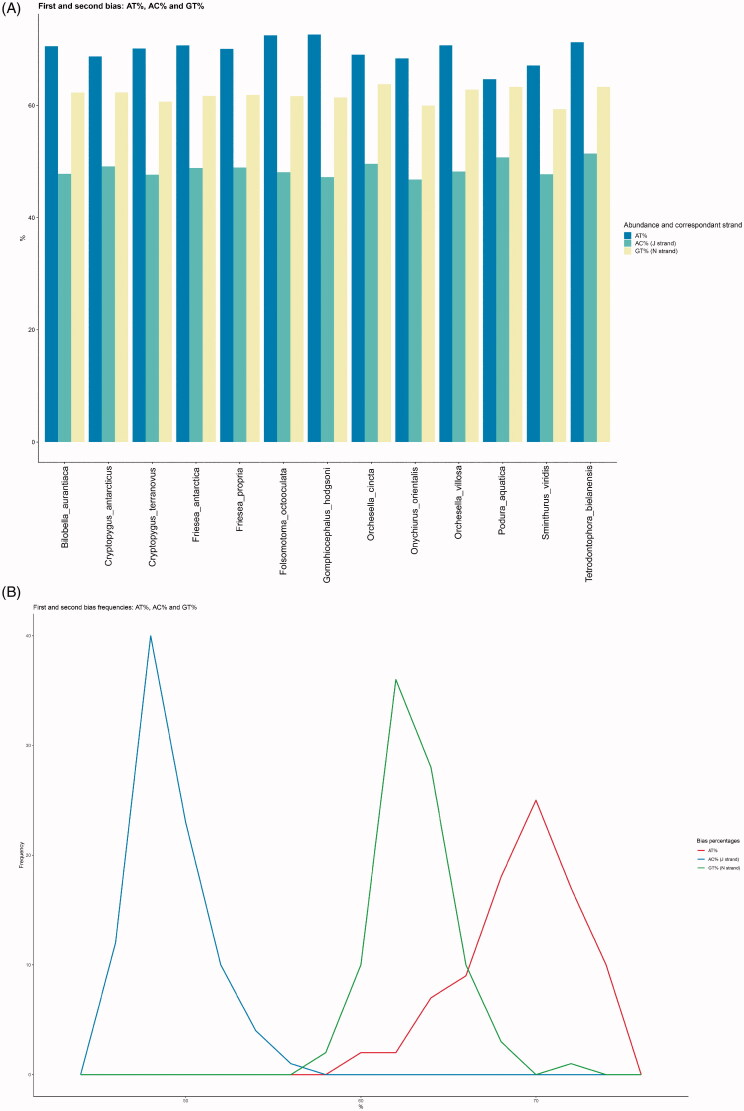
EZskew graphical output of genome biases. (A) AT%, AC%, and GT% by genome with ≤20 genomes. (B) frequency distribution of AT%, AC%, and GT% with >20 genomes.

**Figure 2.**
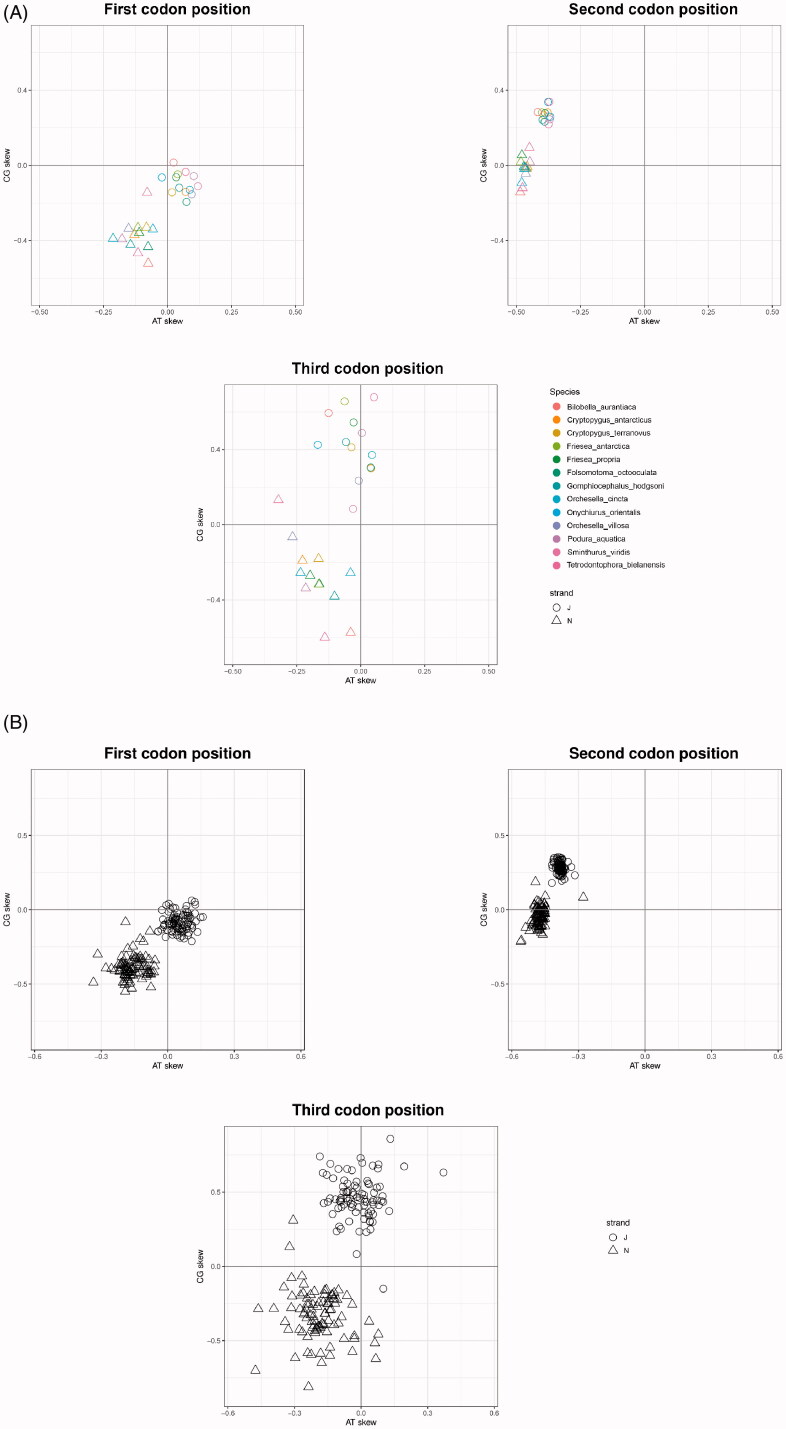
EZskew graphical output of codon biases. (A) CG vs. AT skew by codon position with species color-coded (≤20 genomes). Strand is indicated using shapes. (B) CG vs. AT skew by codon position (>20 genomes). Strand is indicated using shapes.

Base compositional biases were visualized using EZskew for both datasets. Calculations required 15 s for the XL data set and 8 s for the S data set (Table 1). Graphical output automatically adapted the level of detail depending on the number of genomes analyzed. The S dataset (≤20 genomes) produced base composition plots as histograms by genome (Figure 1(A)) and skew plots with individual genomes (i.e. species) identified using colors (Figure 2(A)). The XL dataset (>20 genomes) produced base composition graphics as frequency polygons plots (Figure 1(B)) and skew plots with individual genomes not individually identified (Figure 2(B)).

### 3.3. EZcodon output

**Figure 3.**
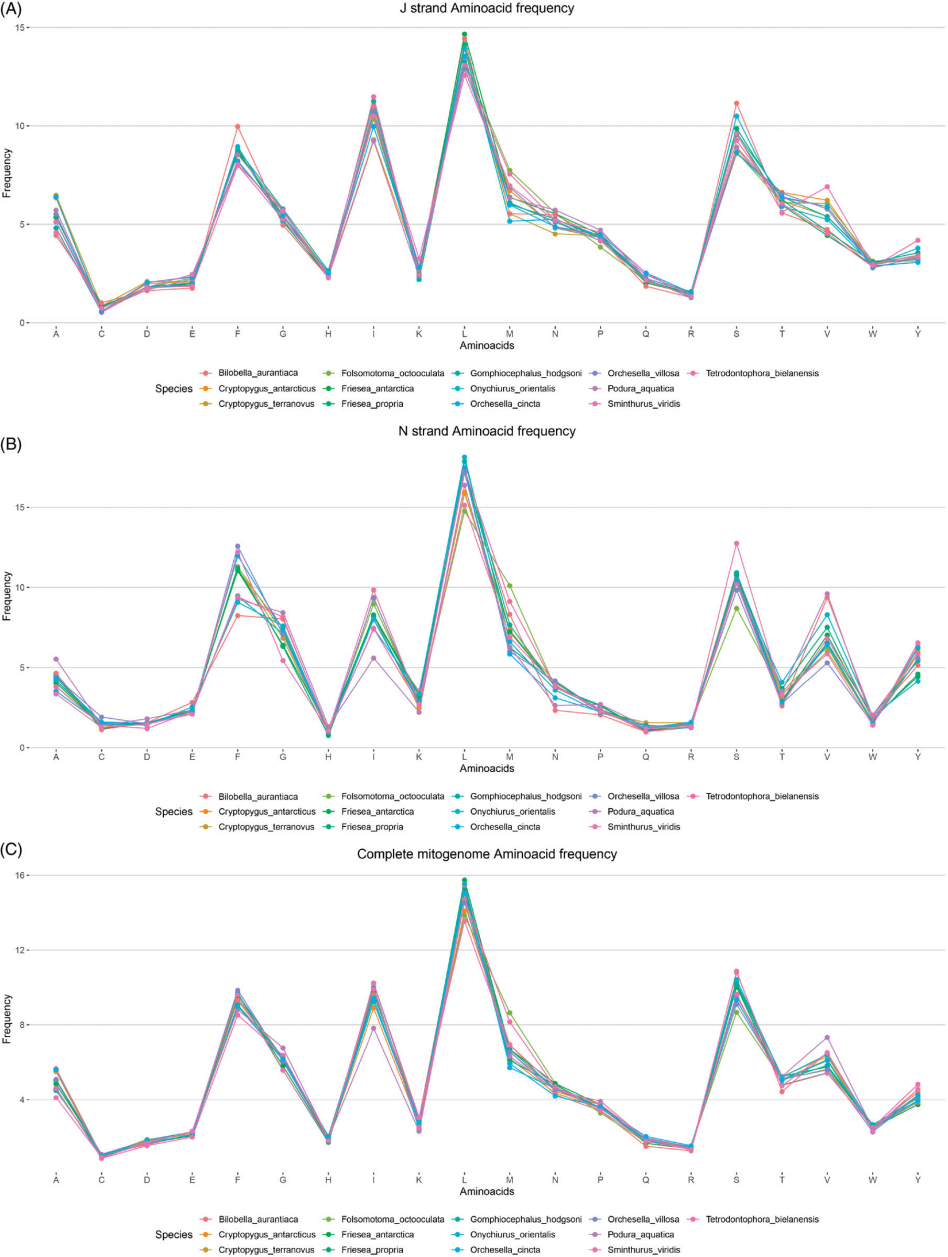
EZcodon graphical output. Amino acid frequencies by genome (≤20 genomes) and by strand (J strand only, N strand only, full data). Genomes are color-coded.

**Figure 4.**
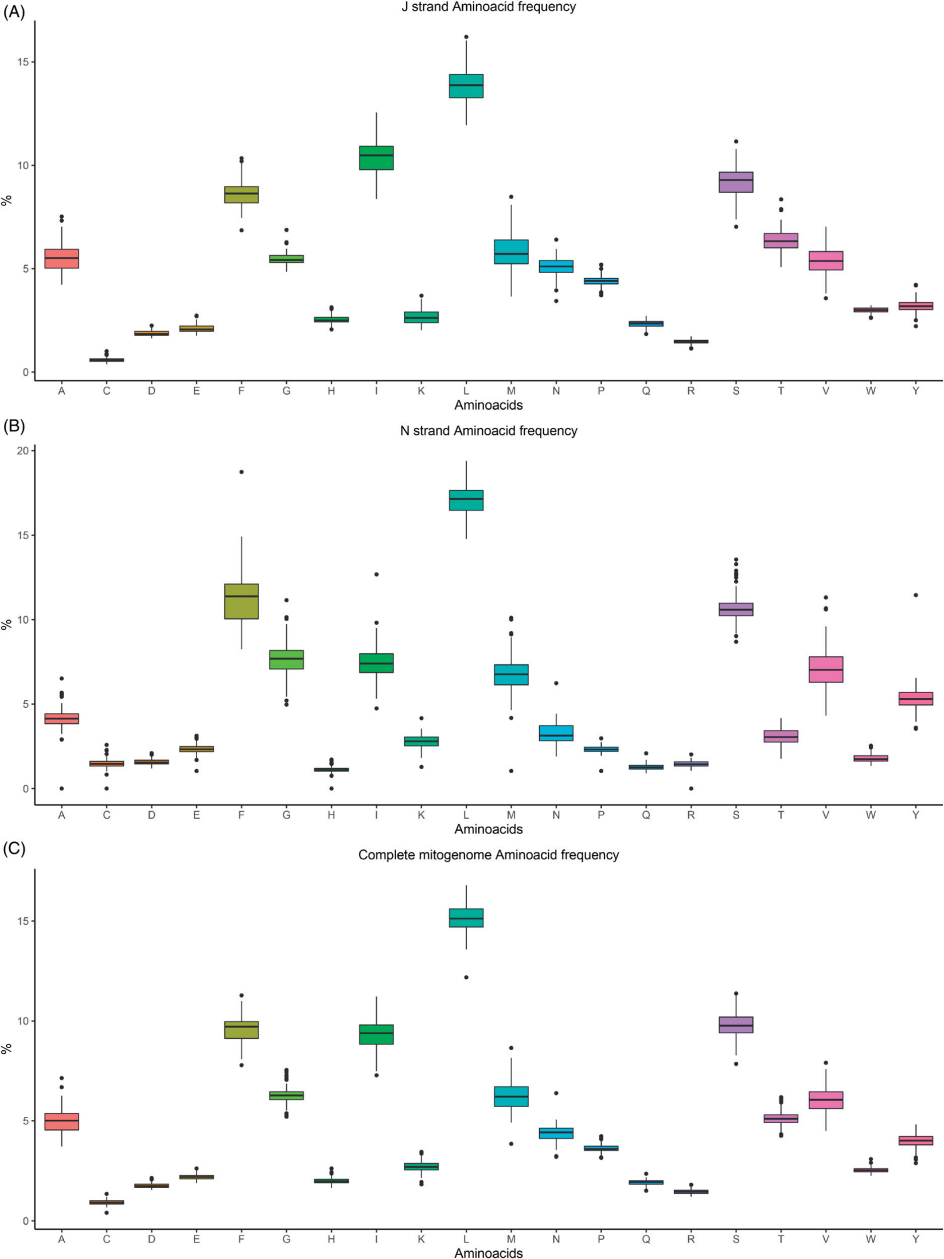
EZcodon graphical output. Amino acid frequencies as boxplots (>20 genomes), by strand (J strand only, N strand only, full data).

**Figure 5.**
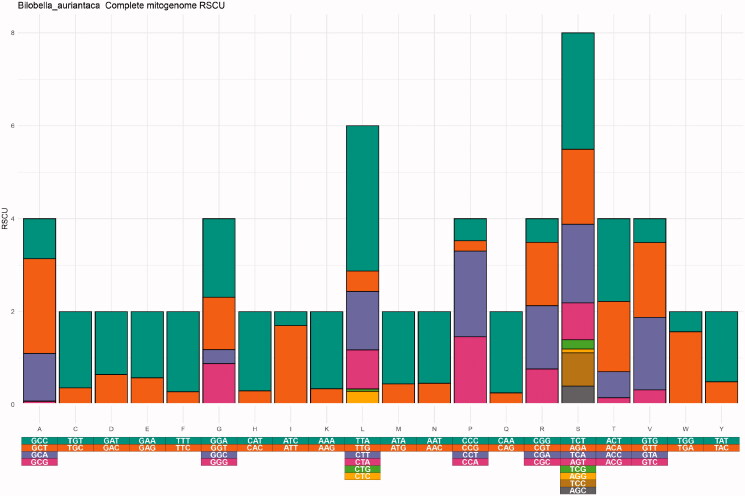
EZcodon graphical output. RSCU by genome, only one genome is displayed. Codons are color-coded.

Amino acid frequencies and codon usage were visualized using EZcodon. Analysis of the S data set required 23 s, while the XL data set required 110 s (Table 1). EZcodon generated different plots based on the number of genomes analyzed. Amino acid frequency plots resulted in a linear histogram with individual genomes identified in color (Figure 3) for the S dataset (≤20 genomes) and a series of boxplots (Figure 4) for the XL data set (>20 genomes). A different RSCU plot was produced for each genome being analyzed (displayed as Figure 5 for one single genome).

## 4. Discussion

Due to the relative ease in sample preparation and the decreasing costs for sequencing, complete mitochondrial genome sequences are being produced at an unprecedented rate and phylomitogenomic analyses are used more and more frequently to address phylogenetic issues at different taxonomic levels. Based on our laboratory common practices for data analysis, we developed EZmito, a simple and intuitive bioinformatic tool capable of automating some basic data preparation and visualization steps. This is currently implemented as freely usable Web Server. All functions are relatively fast, with even large data sets (e.g. with 90 taxa) being processed in less than 2 min. Moreover, EZmito graphic tools (EZskew and EZcodon) produce the most commonly employed graphics as publication-quality images as well as tabular outputs for users willing to customize their plots further. These two tools adapt the level of detail to the number of genomes being analyzed to produce easily readable and quality output.

EZpipe does not include a tree-building step but produces files that can be readily analyzed using common software or submitted to alternative freely available web resources such as the CIPRES Science Gateway. Future improvements to EZmito will depend on usage statistics, but other functions – mostly concerning analysis and visualization, not tree building – are planned.

In summary, EZmito cannot compare to extremely effective and wide-ranging software solutions such as PhyloSuite and NGphylogeny in terms of functions or computational efficiency. At variance, the strength of EZmito lays in its ease of use and the possibility, by focusing on a specific type of data and analytical procedure, to provide a guided interface to the user to perform a simple, yet correct, and effective, analysis of a mitochondrial genome dataset.

## Data Availability

The authors confirm that the data supporting the findings of this study are available within the articles available at https://doi.org/10.1007/s00300-019-02466-8 (Carapelli et al. 2019) and https://doi.org/10.3390/genes12010044 (Cucini et al. 2020) and their corresponding supplementary materials.
